# An overview of traditional smoking cessation interventions and E-cigarettes

**DOI:** 10.3389/fphar.2024.1293062

**Published:** 2024-07-22

**Authors:** Fahad S. Alshehri

**Affiliations:** Department of Pharmacology and Toxicology, College of Pharmacy, Umm Al-Qura University, Makkah, Saudi Arabia

**Keywords:** E-cigarettes, smoking cessation, smoking treatment, health effects, NRT

## Abstract

Many people still struggle with quitting smoking despite available treatment options, making it one of the most significant public health challenges that our society faces. The use of electronic cigarettes (E-cigarettes) has become increasingly popular among people who are seeking to quit smoking. The objective of this review paper is to present a comprehensive analysis of the mechanisms, several types, and impact of E-cigarettes, along with supporting evidence indicating their efficacy in aiding smokers to quit tobacco usage. Additionally, the review discusses recent developments in the treatment of smoking cessation, which include conventional smoking cessation methods. Also, the review discusses the challenges, potential risks, ethical considerations, and controversies surrounding the use of E-cigarettes. The present review presents a comprehensive examination of the existing methods and approaches employed in smoking cessation, including the emerging utilization of E-cigarettes as an effective option in smoking cessation. It explores their efficacy as a valuable instrument in promoting smoking cessation.

## Introduction

Smoking is responsible for over seven million deaths annually on a global scale (Office of the Surgeon General (US), 2004). It is associated with numerous health complications, including lung cancer, cardiovascular disease, stroke, and respiratory disorders (Stallones, 2015; Alexandrov et al., 2016; Rahal et al., 2017; Khani et al., 2018; Scherubl, 2022). While many countries have seen a notable decline in smoking rates over recent decades, smoking remains a substantial public health issue, particularly within specific population groups (Reitsma et al., 2021; Dai et al., 2022; van Hoogstraten et al., 2023). Nicotine addiction is associate with several symptoms including relapse, craving, and withdrawal, each of which plays a significant role in sustaining the addictive behavior (Killen et al., 1992; Benowitz, 2008a). Relapse, characterized by the resumption of smoking following cessation attempts, is often precipitated by environmental triggers or stressors (Piasecki et al., 2002). Craving, a central feature of nicotine addiction, includes intense desires to smoke, triggered by cues associated with smoking or periods of nicotine deprivation (Tiffany et al., 2009). Withdrawal symptoms, including irritability, anxiety, cognitive impairment, increased appetite, and sleep disturbances, intensify upon cessation due to the abrupt cessation of nicotine intake (Devi et al., 2023). Smoking cessation is thus a critical public health priority, given the significant morbidity and mortality linked to tobacco use (Rennard and Daughton, 2000; Gallucci et al., 2020). Despite the availability of numerous smoking cessation interventions, their efficacy varies, and many smokers continue to struggle with quitting.

Electronic nicotine delivery systems, commonly referred to as E-cigarettes, have emerged as a viable method for facilitating smoking cessation (Grana et al., 2014). E-cigarettes are battery-powered devices that heat a liquid, usually containing nicotine, into an aerosol that is inhaled by the user (Breland et al., 2017). These devices first appeared in the early 2000s and have since gained popularity among smokers seeking an alternative to conventional tobacco cigarettes (NIJ, 2020). Even with the controversial facts regarding. Research findings suggest that E-cigarettes may be more effective for smoking cessation compared to traditional methods such as NRT or behavioral counseling (Bullen et al., 2013; Caponnetto et al., 2013; Wise, 2013; McRobbie et al., 2014; Hartmann-Boyce et al., 2021). However, concerns have been raised about the safety and long-term health effects of E-cigarettes. Additionally, there is apprehension about their potential use by young people as an entry point into tobacco use (Kaisar et al., 2016; Ghosh and Drummond, 2017).

Despite these concerns, E-cigarettes have gained popularity as a smoking cessation tool. Early E-cigarettes were often marketed as a way to enjoy the experience of smoking without the negative health effects of traditional cigarettes (CASAA, 2023). However, these early devices were often unreliable and inconsistent, raising concerns about the safety and quality of the liquid solutions used (Laugesen, 2008). Initially, E-cigarettes were introduced as consumer products, which allowed them to be marketed as lifestyle products, which bypass regulatory agencies regulations (Aaron, 2021). However, as evidence of potential risks grew, regulatory agencies began to take action. In 2016, the FDA extended its authority over E-cigarettes through the Deeming Rule, classifying them as tobacco products (Tilburg et al., 2017; Fulmer, 2021; Moysaenko, 2023). This required manufacturers to submit premarket applications, disclose ingredients, include warning labels, and implement youth access restrictions, ensuring consistent quality and safety standards (Barraza et al., 2017).

Currently, no E-cigarettes are approved by the FDA as smoking cessation devices or authorized to make modified risk claims due to several reasons. Manufacturers have not provided sufficient long-term evidence proving safety and effectiveness (Hampsher-Monk et al., 2024; Lindson et al., 2024). Concerns about nicotine addiction, harmful chemicals, and unknown long-term health effects persist (Bhatt et al., 2020; Yammine et al., 2023). Additionally, the rise in adolescent e-cigarette uses and the potential for these devices to lead to traditional smoking have increased the public health concerns. These factors contribute to the FDA’s for not approving e-cigarette for smoking cessation.

On the other hand, E-cigarettes have undergone significant changes and improvements, including the introduction of more reliable devices, improved battery technology, and a wider range of flavors and nicotine strengths (CASAA, 2023). However, concerns about the safety and health effects of E-cigarettes have continued to grow, and the debate over their role in smoking cessation and harm reduction remains ongoing (Wang et al., 2019; Cao et al., 2021). E-cigarettes remain a popular alternative to traditional cigarettes, and their role in smoking cessation and harm reduction continues to be an active area of research (Rom et al., 2015). The objective of this review article is to provide a comprehensive examination of existing smoking cessation approaches while also exploring the possible use of E-cigarettes in this context and assessing their effectiveness and adverse effects as a means for quitting smoking.

This narrative review systematically examines the current literature investigating the traditional smoking cessation interventions and the utilization of E-cigarettes. The critical assessment encompasses a diverse range of studies, including but not limited to clinical trials, observational, and interventional research for smoking cessation. The literature search was methodically created to employ key terms relevant to smoking cessation methods and electronic cigarette interventions. Recognizing the interpretative nature of a narrative review, this review presents a structure to provide a comprehensive overview, beginning with an exploration of historical perspectives on traditional smoking cessation and E-cigarettes. Subsequent sections investigate into current research, categorized by methodological approaches, recent developing treatment, and interventions in smoking cessation.

## Conventional smoking cessation methods

**FIGURE 1:**
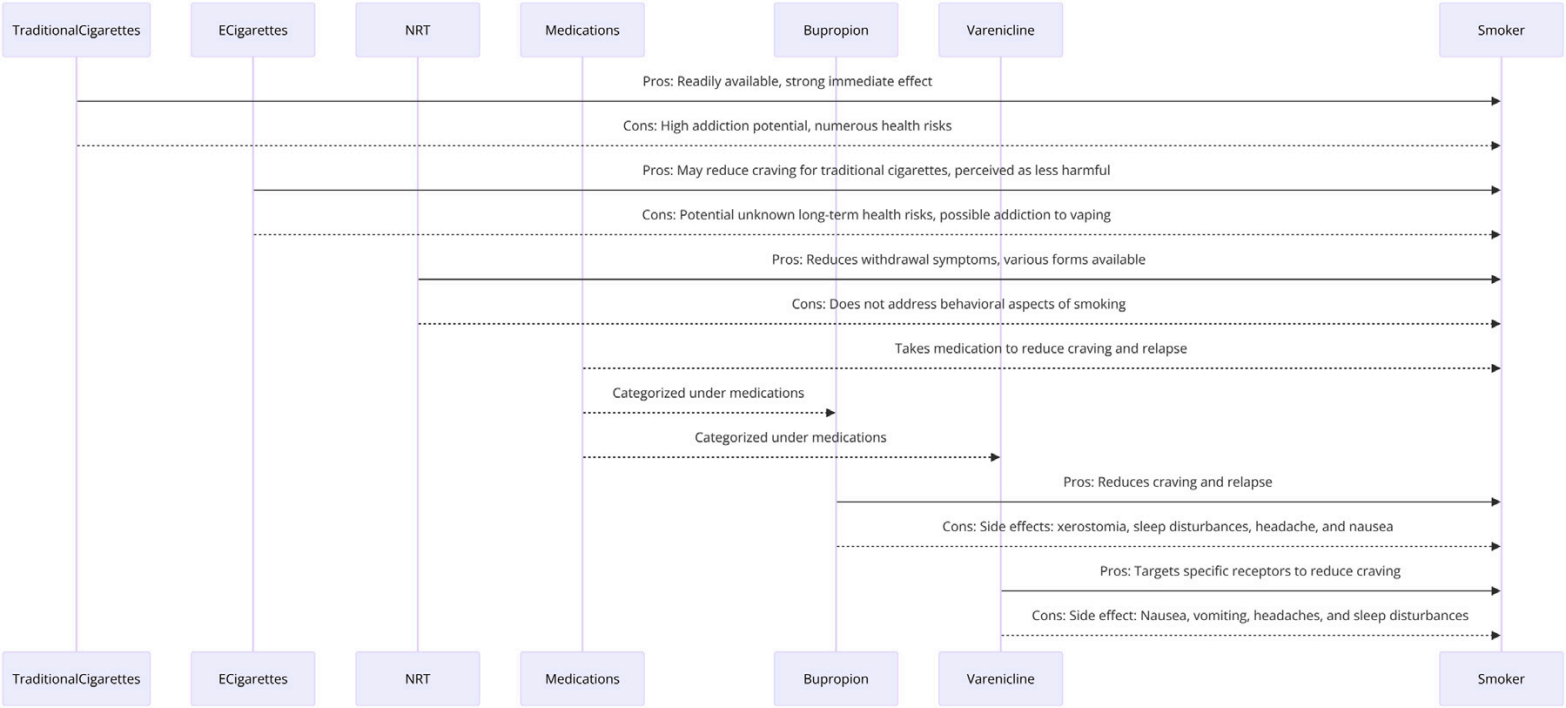
Summary of current smoking forms and current treatment options.

There are several methods available, including NRT and prescription medications (Figure 1).

### Nicotine replacement therapy (NRTs)

Nicotine is the primary addictive component of cigarettes and E-cigarettes, produce its effects on the brain’s reward system, leading to addiction (Herman and Tarran, 2020). This process begins with the binding of nicotine to nicotinic acetylcholine receptors (nAChRs) in the brain (Wittenberg et al., 2020). Activation of these receptors stimulates the release of neurotransmitters such as dopamine, which plays a key role in reinforcing addictive behaviors (Faure et al., 2014). Dopamine release in response to nicotine creates a pleasurable sensation, reinforcing the association between smoking and reward (Benowitz, 2008a). Therefore, chronic nicotine exposure leads to neuroadaptations in the brain, including changes in receptor sensitivity and neurotransmitter levels, which contribute to the development of tolerance and dependence (Markou, 2008). Over time, individuals require increasing amounts of nicotine to achieve the same effects, leading to continued use and addiction (Benowitz, 2008b).

NRT purposes to alleviate cravings and withdrawal symptoms by providing a low dose of nicotine to help reduce withdrawal symptoms (Molyneux, 2004). NRTs are effective in helping smokers quit (Hartmann-Boyce et al., 2018); however, their success rates are relatively modest, with only around 10% of users quitting smoking for more than 6 months due to adherence issues (Mersha et al., 2020). NRT products is designed to help alleviating the withdrawal symptoms while gradually reducing nicotine dependence. By providing a steady supply of nicotine, NRT helps to satisfy cravings and reduce the urge to smoke, thereby supporting individuals in their cessation efforts. Additionally, NRT allows individuals to gradually reduce their nicotine intake, facilitating a smoother transition to abstinence. NRTs, such as nicotine gum, patches, inhalers, and lozenges, are FDA-approved smoking cessation aids (Leelavathi, 2019).

Nicotine patches represent an additional variant of NRT, offering a consistent and regulated supply of nicotine via dermal absorption (Rigotti et al., 2022). The patches are available in different strengths, 7, 14, and 21 mg, and are designed to release nicotine over 24 h (Mendelsohn, 2013). Nicotine patches work by gradually reducing the level of nicotine in the body, thereby reducing the severity of withdrawal symptoms associated with nicotine addiction (Wadgave and Nagesh, 2016). One of the benefits of nicotine patches is their convenience to users, as they require only one application per day. Common side effects of nicotine patches may include skin irritation, itching, or redness at the site of application. Some people may also experience dizziness, headaches, or upset stomach (Wisborg et al., 2000; Weinberger et al., 2014).

Nicotine gum is a type of NRT that delivers nicotine to the body through the lining of the mouth (Garvey et al., 2000). The gum is designed to be chewed and releases nicotine into the bloodstream as it is absorbed through the tissues in the buccal mucosa in the mouth (Schneider et al., 1983). The gum is available in various flavors, 2 and 4 mg, depending on the individual’s level of nicotine dependence (Shiffman et al., 2009). Common side effects include a tingling sensation in the mouth, hiccups, and indigestion. In rare cases, people may experience an allergic reaction to the gum, resulting in symptoms such as difficulty breathing, rash, or swelling (Herrera et al., 1995).

Nicotine lozenges are another form of NRT that delivers nicotine to the body through the lining of the mouth as nicotine gum (Shiffman, 2007). The lozenges are available in different strengths, 2 and 4 mg, depending on the individual’s level of nicotine dependence (Shiffman et al., 2002). They are also available in various flavors, such as mint, cherry, and orange to help users overcome the bitterness of nicotine taste (Terrie, 2010). The purpose behind the formulation of the lozenges is to facilitate a gradual dissolution process within the oral cavity, enabling the absorption of nicotine through the buccal mucosa tissues, thus facilitating its entry into the bloodstream. Common side effects of nicotine lozenges may include a tingling or burning sensation in the mouth, sore throat, nausea, or hiccups (Schnoll et al., 2010).

### Medications

There are two FDA-approved medications for smoking cessation: bupropion (Zyban) and varenicline (Chantix), (Figure 1). Both medications work by reducing nicotine cravings and withdrawal symptoms. Bupropion is a medication that is used in smoking cessation as a form of pharmacotherapy (Richmond and Zwar, 2003). Bupropion inhibits the reuptake of dopamine and norepinephrine, therefore, modulating mood changes, and reduce seeking behavior of smoking (Foley et al., 2006). Dopamine and norepinephrine are neurotransmitters play key roles in the brain’s reward and reinforcement pathways, making them central to nicotine addiction (Fowler et al., 2020). Through the elevation of neurotransmitter levels within the brain, bupropion effectively diminishes the desire for nicotine and mitigates the various symptoms experienced during the process of quitting smoking (Shiffman et al., 2000). This can reduce cravings and withdrawal symptoms, through of dopamine and norepinephrine signaling which counteracts the dysregulated neurotransmitter systems associated with nicotine addiction (United States Public Health Service Office of the Surgeon General, 2020). By restoring balance to these systems, bupropion helps to alleviate cravings and withdrawal symptoms, making it easier for individuals to quit smoking (Yan and Goldman, 2021). Thus, Bupropion is usually prescribed as a 12-week course of treatment, during which time the individual gradually reduces cigarette consumption before quitting completely (Simon et al., 2004). Typical adverse reactions associated with the use of bupropion encompass symptoms such as xerostomia, sleep disturbances, headache, and nausea (Wilkes, 2008).

Varenicline is a prescription medication used as a smoking cessation aid (Garrison and Dugan, 2009). Varenicline stimulates nicotine receptors α7 nicotinic acetylcholine receptors, mimicking the effects of nicotine and partial agonist on the α4-β2, α3-β4, and α6-β2 (Niaura et al., 2006). The α4β2 receptors are highly sensitive to nicotine and are involved in the release of dopamine (McCaul et al., 2020). Varenicline’s partial agonist activity means it binds to these receptors and activates them, but to a lesser degree than nicotine (Papke, 2024). This partial stimulation helps to attenuate cravings and withdrawal symptoms by providing a moderate level of receptor activation, reducing the urge to smoke without producing the same level of dopamine release as nicotine, thereby lowering the potential for addiction (Ebbert et al., 2015). This action reduces cravings for nicotine and reduces withdrawal symptoms (Brandon et al., 2011). Simultaneously, varenicline obstructs the outcomes of nicotine in the event that an individual engages in smoking, thereby diminishing the pleasure derived from the act and creating a diminished inclination to endure in such behavior (Brandon et al., 2011). Typically, the duration of varenicline therapy spans a period of 12 weeks, although an extended treatment period of 12 weeks can be considered for those individuals who have effectively ceased their smoking habit (Pachas et al., 2012). Varenicline is more effective than other smoking cessation medications, such as NRT and bupropion (Aubin et al., 2008; West et al., 2008). It is important to note that varenicline can cause a number of potential side effects, such as nausea, vomiting, headaches, and sleep disturbances (McClure et al., 2009).

## E-cigarettes for smoking cessation

Extensive research has been conducted on E-cigarettes in relation to smoking cessation, and the findings regarding their effectiveness in comparison to alternative interventions present a combination of results (Rigotti, 2020; Hartmann-Boyce et al., 2023) Supplementary Table S1. Several reports have found that E-cigarettes could be more effective than traditional NRT, such as nicotine patches or gum, while others have found no difference between the two. For example, a randomized controlled trial investigating E-cigarettes over 13 weeks, with or without nicotine, found a potential effect in aiding smoking cessation, with minimal adverse consequences. The results were comparable to those achieved through the usage of nicotine patches. Additionally, the study revealed that nicotine-infused E-cigarettes demonstrated superior efficacy compared to both placebos and patches in facilitating the cessation of smoking. However, it is worth noting that these disparities failed to attain statistical significance (Bullen et al., 2013).

In another randomized controlled trial, 886 participants were assigned to either an e-cigarette or a nicotine replacement group. In the nicotine-replacement group, the 1-year abstinence rate was observed to be 9.9%, whereas the e-cigarette group exhibited a comparatively higher rate of 18.0%. Individuals who successfully abstained from using tobacco products within the E-cigarette group demonstrated a higher tendency to persist with their designated product after a duration of 52 weeks. Furthermore, the E-cigarette group exhibited a more reduction in coughing and the production of phlegm in comparison to the nicotine-replacement group. Nevertheless, notable differences between the groups were not observed in terms of the prevalence of wheezing or shortness of breath (Hajek et al., 2019).

Furthermore, an additional randomized controlled trial conducted a comparative assessment of the efficacy of E-cigarettes in comparison to nicotine gum for promoting smoking cessation. The study yielded results indicating that there were no statistically notable variances in the rates of abstinence at different time points. However, it was observed that the group utilizing E-cigarettes exhibited a greater percentage of participants who experienced a decrease in smoking after 24 weeks when compared with the group utilizing nicotine gum. Additionally, the E-cigarette group reported a lower occurrence of side effects compared to the nicotine gum group. Consequently, the findings of this study imply that E-cigarettes have the potential to serve as an effective form of nicotine replacement therapy for individuals aiming to quit smoking. (Lee et al., 2019).

This may make E-cigarettes more satisfying for smokers, leading to higher rates of quit success. Additionally, E-cigarettes can be customized with assorted flavors and nicotine strengths, allowing smokers to tailor their use to their individual preferences. Nevertheless, it is crucial to acknowledge the limitations associated with E-cigarettes, which can impede their efficacy for all individuals. These findings suggest that E-cigarettes might not serve as a reliable cessation aid for adult smokers and could potentially foster nicotine addiction (Chen et al., 2020).

## Safety and ethical concerns for E-cigarettes

E-cigarettes have been advertised as a safer alternative to traditional cigarettes, but there are concerns about their safety as a cessation tool and may help some smokers quit, (Wollscheid and Kremzner, 2009; Franck et al., 2016; Drazen et al., 2019). One concern is that E-cigarettes may expose users to harmful chemicals and toxins, including heavy metals, volatile organic compounds, and ultrafine particles, which can have negative health effects (Rahman et al., 2014). Furthermore, the impacts on health resulting from the utilization of E-cigarettes remain uncertain. Furthermore, certain indications imply that E-cigarettes could potentially heighten the vulnerability to pulmonary ailments and cardiovascular condition (Sapru et al., 2020). Additionally, there exists the possibility that E-cigarettes may function as an entry point to traditional cigarette use, particularly among young people (Jankowski et al., 2019). This could potentially lead to a new generation of nicotine users and contribute to the overall burden of tobacco-related disease (Leventhal et al., 2019).

The putative toxicological effects associated with e-cigarette use is a topic of significant concern and ongoing investigation. Preclinical studies, encompassing cell culture and animal models, have contributed valuable insights into the potential health implications of e-cigarette aerosols (Kalininskiy et al., 2021). These studies suggest that the inhalation of e-cigarette vapor may expose users to a range of harmful constituents, including heavy metals, volatile organic compounds, and ultrafine particles (Eaton et al., 2018; Papaefstathiou et al., 2020). Clinical studies further explore the impact of e-cigarette use on human health, with findings indicating potential respiratory and cardiovascular effects (Marques et al., 2021). The World Health Organization (WHO) underscores the need for caution, emphasizing that the long-term health effects of E-cigarettes remain uncertain and warrant thorough evaluation (Yong et al., 2017; Yagi et al., 2023). WHO recommends strict regulatory measures to address the marketing, sale, and use of E-cigarettes, particularly among youth (Yagi et al., 2023). Additionally, WHO encourages comprehensive research efforts to better understand the potential risks associated with e-cigarette use and its implications for public health (Chen-Sankey and Bover-Manderski, 2022). As the scientific community attempts to understand the complexities of e-cigarette toxicology, adherence to WHO guidelines is essential for the public health safety (Gordon et al., 2022).

Therefore, it is important to promote e-cigarette use as a last resort and encourage individuals to try other proven smoking cessation methods first (Thirlway, 2016). There is a risk of E-cigarettes being marketed to non-smokers, particularly youth, as a trendy and harmless alternative to traditional cigarettes. This could lead to an increase in nicotine addiction and smoking behavior, which could have negative long-term health consequences.

## Expert recommended opinions in smoking cessation and E-cigarette utilization

The scientific community is engaged in ongoing research and discussions regarding the comparative efficacy and safety of traditional smoking cessation methods and E-cigarettes. Traditional methods, such as nicotine NRT and prescription medications, have been studied extensively and are endorsed by health organizations worldwide. NRT, including patches, gum, lozenges, and prescription medications like bupropion and varenicline, has shown effectiveness in aiding smoking cessation, even though with modest success rates. On the other hand, E-cigarettes have emerged as a novel approach to smoking cessation, generating significant interest and controversy. Some studies suggest that E-cigarettes may offer a more appealing and customizable alternative for smokers, potentially contributing to higher quit rates compared to traditional methods. However, concerns persist regarding the safety of E-cigarettes, particularly regarding exposure to harmful chemicals and long-term health effects.

While several reports have found no significant differences between conventional nicotine replacement therapies (NRT) like nicotine patches or gum and E-cigarettes in terms of smoking cessation outcomes, there are specific areas where NRT may offer more effective results or distinct advantages. Nicotine patches and gum have been tested and approved by regulatory agencies such as FDA for smoking cessation. These products have standardized dosages and quality controls, ensuring consistent delivery of nicotine, which enhances their reliability and safety profiles. On the other hand, the variability in e-cigarette devices and formulations can lead to inconsistent nicotine delivery. In addition, NRT products, including patches and gum, have a well-documented safety profile with minimal side effects. These products do not involve inhaling vaporized substances, eliminating exposure to potentially harmful chemicals found in e-cigarette aerosols, such as volatile organic compounds and heavy metals. This makes NRT a safer option, particularly for individuals with respiratory conditions or those concerned about long-term health effects. Also, NRT aims exclusively at smoking cessation and does not replicate the behavioral aspects of smoking, which can help reduce the risk of dual use.

## Conclusion

Smoking cessation is an important public health issue that requires ongoing attention and effort. There exists a range of available strategies to aid individuals in quitting smoking, encompassing pharmaceutical interventions and alternative therapies such as E-cigarettes. Each approach possesses its own advantages and limitations, thus necessitating treatment options customization to align with each patient’s specific needs and preferences. E-cigarettes have emerged as a subject of controversy when considered as a means of smoking cessation. This is due to fears surrounding their safety and effectiveness. Although the body of research on the efficacy of E-cigarettes as a smoking cessation tool remains limited, E-cigarettes have become a recognized as an effective option to help people in quitting smoking. Nevertheless, healthcare providers must carefully evaluate the potential advantages and drawbacks associated with E-cigarettes and ensure that their use is grounded in the latest evidence and guidelines.

## Supplementary material

SUPPLEMENTARY TABLE S1 Recent clinical trials on E-cigarettes as a tool for smoking cessation.
