# Supplementary material for: An overview of traditional smoking cessation interventions and E-cigarettes
Source: Front Pharmacol. 2024 Jul 22;15:1293062. doi: 10.3389/fphar.2024.1293062 (PMC11298375; doi:10.3389/fphar.2024.1293062)
Supplement: Supplementary file 1 [file Table1.pdf]

| # | Study Title | Study Status | Conditions | Interventions | Sponsor | Collaborators | Study Type |
|---|---|---|---|---|---|---|---|
| 1. | Efficacy and Safety of E-cigarettes for Smoking Cessation in Middle-aged Heavy Smokers | Completed | Smoking Cessation\|Electronic Cigarette | DRUG: Nicotine\|DRUG: Varenicline Tartrate\|BEHAVIORAL: Motivational Interview | University of Oulu | Lapland Central Hospital Rovaniemi Finland | Interventional |
| 2. | Enhanced E-cigarette Coaching Intervention for Dual Users of Cigarettes and E-cigarettes | Completed | Smoking Cessation | BEHAVIORAL: Enhanced E-cigarette Coaching\|BEHAVIORAL: Quitline treatment as usual | Consumer Wellness Solutions | University of Oklahoma\|SRI International\|H. Lee Moffitt Cancer Center and Research Institute | Interventional |
| 3. | A Structured Protocol to Evaluate Efficacy and Safety of a Popular Electronic Nicotine Delivery Device (E-Cigarette) Efficacy and Safety of a Popular Electronic Nicotine Delivery Device (E-Cigarette) | Completed | Healthy Smokers\|Smoking Cessation | | Universita degli Studi di Catania | Lega Italiana Anti Fumo\|Arbi Group Srl | Observational |
| 4. | Implementation and Dissemination of an Evidence-Based Tobacco Product Use Prevention Videogame | Completed | Smoking Cessation\|Smoking | DEVICE: smokeSCREEN web-based game | Yale University | | Interventional |

| | | | | | | | |
|---|---|---|---|---|---|---|---|
| | Intervention With Adolescents | | | | | | |
| 5. | The ESTxENDS Trial-Electronic Nicotine Delivery Systems as an Aid for Smoking Cessation-extension of Follow-up | Completed | Smoking Cessation | OTHER: ENDS (vaporizer/e-cig) and smoking cessation counseling\|OTHER: Smoking cessation counseling | University of Bern | University of Lausanne\|University of Geneva, Switzerland\|University of Zurich\|State Hospital, St. Gallen\|Swiss National Science Foundation\|Krebsforschung Schweiz, Bern, Switzerland\|Federal Office of Public Health, Switzerland | Interventional |
| 6. | Nicotine Pharmacokinetics From Research Electronic Nicotine Delivery System S-TA-U001 in Smokers and E-Cigarette Users | Completed | Cigarette Smoking | DRUG: Research ENDS S-TA-U001\|DRUG: Nicorette Inhalator | NJOY, Inc. | LA Clinical Trials\|National Institute on Drug Abuse (NIDA) | Interventional |
| 7. | Early Smoking Reduction or Cessation by Means of no Nicotine Electronic Cigarette Added to Standard Counselling. | Completed | Cancer\|Myocardial Infarction | DEVICE: No nicotine electronic cigarette\|OTHER: Standard counseling | European Institute of Oncology | | Interventional |

| | | | | | | | |
|---|---|---|---|---|---|---|---|
| 8. | Does Abstinence From E-cigarettes Produce Withdrawal Symptoms? | Completed | Substance Withdrawal Syndrome | BEHAVIORAL: Abstinence from e-cigarettes | University of Vermont | National Cancer Institute (NCI) | Interventional |
| 9. | Evaluating the Efficacy of E-Cigarette Use for Smoking Cessation (E3) Trial | Completed | Smoking Cessation | OTHER: Nicotine E-Cigarette\|OTHER: Non-Nicotine E-Cigarette\|BEHAVIORAL: Counseling | McGill University | Sir Mortimer B. Davis - Jewish General Hospital | Interventional |
| 10. | Quit IT: Preliminary Testing of a Web-based, 3D Coping Skills Game to Increase Quitting Self-Efficacy for Maintaining Smoking Abstinence Following Hospitalization | Completed | Tobacco Use Cessation | OTHER: web-based video game (Quit IT)\|BEHAVIORAL: Questionnaires\|OTHER: Salvia sample | Memorial Sloan Kettering Cancer Center | New York University\|Muzzy Lane Software | Interventional |
| 11. | ESTxENDS Trial-Substudy on Oxidative Stress Induced by Electronic Nicotine Delivery Systems (ENDS) Measured in Urine | Completed | Smoking Cessation\|Oxidative Stress | OTHER: ENDS (vaporizer/e-cig) and smoking cessation counseling\|OTHER: smoking cessation counseling | University of Bern | University of Lausanne\|University of Geneva, Switzerland\|University of Zurich\|State Hospital, St. Gallen\|Swiss National Science Foundation\|Krebsforschung Schweiz, Bern, Switzerland\|Federal | Interventional |

| | | | | | | | |
|---|---|---|---|---|---|---|---|
| | | | | | | Office of Public Health, Switzerland | |
| 12. | Clinical Learning Study for a Mobile Smoking Cessation Program | Completed | Smoking Cessation\|Smoking Behaviors\|Smoking Reduction\|Smoking, Cigarette\|Smoking\|Nicotine Dependence | DEVICE: ClickotineÌ® | Click Therapeutics, Inc. | | Observational |
| 13. | Promoting Smoking Cessation in the Community Via QTW 2014 | Completed | Smoking Cessation Intervention | BEHAVIORAL: Quit immediately (QI) / cut down to quit (CDTQ) | The University of Hong Kong | Hong Kong Council on Smoking and Health | Interventional |
| 14. | Efficacy and Safety of an Electronic Nicotine Delivery Device (E-Cigarette) Without Nicotine Cartridges | Completed | Healthy Smokers\|Smoking Cessation | | Universita degli Studi di Catania | Lega Italiana Anti Fumo\|Arbi Group Srl | Observational |
| 15. | The END Perioperative Smoking Pilot Study | Completed | Smoking\|Nicotine Addiction\|Surgery | BEHAVIORAL: ENDD (NJOY)\|DRUG: NRT (NicoDerm CQ)\|BEHAVIORAL: telephone counseling\|BEHAVIORAL: brief advice\|BEHAVIORAL: brochure | University of California, San Francisco | | Interventional |
| 16. | Smoking Cessation and Reduction With an Electronic Nicotine Delivery Device (ENDD) | Completed | Nicotine Dependence\|Smoking Cessation | DEVICE: E-Cigarette 7.2 mg nicotine | Universita degli Studi di Catania | Lega Italiana Anti Fumo\|Arbi Group Srl | Interventional |
| 17. | A Comparison of the Effectiveness of | Completed | Smoking | BEHAVIORAL: quit immediately | The University of Hong Kong | | Interventional |

| | | | | | | | |
|---|---|---|---|---|---|---|---|
| | Two Approaches in Achieving Smoking Abstinence | | | (QI)\|BEHAVIORAL: cut down to quit (CDTQ) | | | |
| 18. | Zonisamide/Bupropion Effects on Switching to Electronic Cigarettes | Completed | Smoking Cessation | DRUG: Zonisamide\|DRUG: Bupropion\|OTHER: Halo G6 e-cigarette | Rose Research Center, LLC | Foundation for a Smoke Free World INC | Interventional |
| 19. | The ESTxENDS Trial-Substudy on the Effects of Using Electronic Nicotine Delivery Systems (ENDS/Vaporizer/E-cig) on Respiratory Symptoms | Completed | Smoking Cessation\|Respiratory Disease | OTHER: ENDS (vaporizer/e-cig) and smoking cessation counseling\|OTHER: Smoking cessation counseling | University of Bern | University of Lausanne\|University of Geneva, Switzerland\|University of Zurich\|State Hospital, St. Gallen\|Swiss National Science Foundation\|Krebsforschung Schweiz, Bern, Switzerland\|Federal Office of Public Health, Switzerland | Interventional |
| 20. | Efficacy and Safety of an Electronic Nicotine Delivery Device (E-Cigarette) | Completed | Healthy Smokers\|Smoking Cessation | | Universita degli Studi di Catania | Lega Italiana Anti Fumo\|Arbi Group Srl | Observational |
| 21. | The Use of Nicotine Patches Together With E-cigarettes (With and Without Nicotine) for Smoking Cessation | Completed | Smoking Cessation | DRUG: Nicotine patch\|DEVICE: e-cigarette\|DRUG: Nicotine\|BEHAVIORAL: Behavioural support | University of Auckland, New Zealand | Health New Zealand Ltd, Christchurch, New Zealand\|Auckland District Health Board | Interventional |
| 22. | The ESTxENDS Trial-Electronic Nicotine | Completed | Smoking Cessation | OTHER: ENDS (vaporizer/e-cig) and | University of Bern | University of Lausanne\|University | Interventional |

| | | | | | | | |
|---|---|---|---|---|---|---|---|
| | Delivery Systems (ENDS/Vaporizer/E-cigarette) as an Aid for Smoking Cessation. | | | smoking cessation counseling\|OTHER: Smoking cessation counseling | | of Geneva, Switzerland\|University of Zurich\|State Hospital, St. Gallen\|Swiss National Science Foundation | |
| 23. | Feasibility of a Decision Aid for E-Cigarettes in Primary Care | Completed | Smoking, Cigarette\|Smoking Cessation | BEHAVIORAL: Smoking Cessation iPad Decision Aid Tool | University of Florida | | Interventional |
| 24. | E-Visit for COPD and Smoking Cessation | Completed | Pulmonary Disease, Chronic Obstructive\|Cigarette Smoking\|Telemedicine | BEHAVIORAL: smoking cessation, early COPD detection, electronic visit\|OTHER: Treatment As Usual | Medical University of South Carolina | | Interventional |
| 25. | ESTxENDS Trial-Substudy on Oxidative Stress Induced by Electronic Nicotine Delivery Systems (ENDS) Measured in EBC | Completed | Smoking Cessation\|Oxidative Stress | OTHER: ENDS (vaporizer/e-cig) and smoking cessation counseling\|OTHER: Smoking cessation counseling | University of Bern | University of Lausanne\|University of Geneva, Switzerland\|Swiss National Science Foundation | Interventional |
| 26. | Randomized Clinical Trial to Reduce Harm From Tobacco | Completed | Smoking Cessation | BEHAVIORAL: E-cigarette free access\|BEHAVIORAL: E-cigarette/NRT/Zyban/Chantix Choice\|BEHAVIORAL: Outcome Incentive arm\|BEHAVIORAL: Loss framing incentive | University of Pennsylvania | Vitality Institute | Interventional |

| | | | | | | | |
|---|---|---|---|---|---|---|---|
| | | | | arm\|BEHAVIORAL: Control | | | |
| 27. | Comparing Nicotine Delivery, Subjective Effects, and Sensory Experiences of Tobacco Users Using Oral Nicotine Products and Electronic Cigarettes [ZYN Study] | Completed | Tobacco Use\|Tobacco Smoking\|Cigarette Smoking\|Smokeless Tobacco Cessation | OTHER: Electronic Cigarette\|OTHER: Oral Nicotine Pouch | Roswell Park Cancer Institute | | Interventional |
| 28. | Preliminary Evaluation of Alternative Approaches to Combustible Cigarette Cessation (Exchange Project Sub-Study) | Completed | Smoking Cessation | BEHAVIORAL: E-cigarette\|BEHAVIORAL: Financial incentives + E-cigarette | University of Oklahoma | | Interventional |
| 29. | The ESTxENDS Trial-Substudy on the Effects of Using Electronic Nicotine Delivery Systems (ENDS) on Sleep Quality. | Completed | Smoking Cessation\|Sleep | OTHER: ENDS (vaporizer/e-cig) and smoking cessation counseling\|OTHER: Smoking cessation counseling | University of Bern | University of Lausanne\|University of Geneva, Switzerland\|University of Zurich\|State Hospital, St. Gallen\|Swiss National Science Foundation\|Krebsforschung Schweiz, Bern, Switzerland\|Federal | Interventional |

| | | | | | | | |
|---|---|---|---|---|---|---|---|
| | | | | | | Office of Public Health, Switzerland | |
| 30. | Smoking Cessation E-Visit | Completed | Smoking | BEHAVIORAL: Smoking cessation e-visit\|BEHAVIORAL: Treatment As Usual | Medical University of South Carolina | | Interventional |
| 31. | E-cigarettes and Cardiovascular Function | Completed | Smoking Cessation\|Cardiovascular Diseases\|Microcirculation\|Macrocirculation | OTHER: Electronic Cigarette and behavioural change support.\|OTHER: Nicotine-Free Electronic Cigarette and behavioural change support.\|OTHER: Nicotine Replacement Therapy and behavioural change support. | Sheffield Hallam University | King's College London\|Heart Research U.K.\|Help2Change | Interventional |
| 32. | Deliver an Evidence-based Smoking Cessation Intervention for Smokers Attending A&E Departments in Hong Kong | Completed | Smoking\|Smoking Cessation\|Evidence-Based Nursing\|Emergency Service, Hospital | BEHAVIORAL: an evidence-based smoking cessation intervention comprising brief advice plus active referrals | The University of Hong Kong | Chinese University of Hong Kong | Interventional |
| 33. | The ESTxENDS Trial-Substudy on the Metabolic Effects of Using Electronic Nicotine Delivery Systems (ENDS/Vaporizer/E-cig) | Completed | Smoking Cessation\|Cardiovascular Diseases | OTHER: ENDS (vaporizer/e-cig) and smoking cessation counseling\|OTHER: Smoking cessation counseling | University of Bern | University of Lausanne\|University of Geneva, Switzerland\|University of Zurich\|State Hospital, St. Gallen\|Swiss National Science | Interventional |

| | | | | | | | |
|---|---|---|---|---|---|---|---|
| | | | | | | Foundation\|Krebsforschung Schweiz, Bern, Switzerland\|Federal Office of Public Health, Switzerland | |
| 34. | UK-Czech E-cigarette Study | Completed | Smoking Cessation | OTHER: Standard care plus electronic cigarettes\|BEHAVIORAL: Standard care | Queen Mary University of London | | Interventional |
| 35. | Smoking Cessation in Women With Gynecological Conditions | Completed | Cervical Dysplasia | OTHER: Nicotine Replacement Therapy\|DEVICE: Electronic Cigarettes | University of Oklahoma | | Interventional |
| 36. | Toxins and Delivery in e-Cigarette Users | Completed | Smoking Cessation | OTHER: e-Cigarette | Queen Mary University of London | | Observational |
| 37. | ESTxENDS Trial: MN Substudy - Micronuclei in Buccal Epithelium, a Surrogate Measure of Future Cancer Risk, Induced by Electronic Nicotine Delivery Systems (ENDS/Vaporizer/E-cig) | Completed | Smoking Cessation\|Micronuclei | OTHER: ENDS (vaporizer/e-cig) and smoking cessation counseling\|OTHER: Smoking cessation counseling | University of Bern | University of Lausanne\|University of Geneva, Switzerland\|University of Zurich\|State Hospital, St. Gallen\|Swiss National Science Foundation\|Krebsforschung Schweiz, Bern, Switzerland\|Federal Office of Public Health, Switzerland | Interventional |
| 38. | Smoking Cessation Self-Help for Dual Users of Tobacco Cigarettes and E-Cigarettes | Completed | Smoking Cessation | BEHAVIORAL: GENERIC\|BEHAVIORAL: eTARGET | H. Lee Moffitt Cancer Center and Research Institute | National Institute on Drug Abuse (NIDA)\|National Institutes of Health (NIH) | Interventional |

| | | | | | | | |
|---|---|---|---|---|---|---|---|
| 39. | The MATCH (Medication Aids for Tobacco Cessation and Health) Study | Completed | Tobacco Use Disorder\|Nicotine Dependence | DRUG: Bupropion\|DRUG: Varenicline\|BEHAVIORAL: Weekly Motivational Emails | Centre for Addiction and Mental Health | Global Research Awards for Nicotine Dependence (GRAND) | Interventional |
| 40. | Combination of E-cigarettes and Varenicline for Tobacco Harm Reduction | Completed | Smoking Cessation\|Harm Reduction | DRUG: Varenicline\|OTHER: e-cigarette | Rose Research Center, LLC | Foundation for a Smoke Free World INC | Interventional |
| 41. | The ESTxENDS Trial-Substudy on Effects of Using Electronic Nicotine Delivery Systems (ENDS) on Olfactory Function | Completed | Smoking Cessation\|Olfactory Disorder | OTHER: ENDS (vaporizer/e-cig) and smoking cessation counseling\|OTHER: Smoking cessation counseling | University of Bern | University of Lausanne\|University of Geneva, Switzerland\|University of Zurich\|State Hospital, St. Gallen\|Swiss National Science Foundation\|Krebsforschung Schweiz, Bern, Switzerland\|Federal Office of Public Health, Switzerland | Interventional |
| 42. | The ESTxENDS Trial-Substudy on the Effect on Toxins From Using Electronic Nicotine Delivery Systems (ENDS/Vaporizer/E-cig) | Completed | Smoking Cessation\|Toxicity | OTHER: ENDS (Vaporizer/e-cig) and smoking cessation counseling\|OTHER: Smoking cessation counseling | University of Bern | University of Lausanne\|University of Geneva, Switzerland\|University of Zurich\|State Hospital, St. Gallen\|Swiss National Science Foundation\|Krebsforschung Schweiz, Bern, | Interventional |

| | | | | | | | |
|---|---|---|---|---|---|---|---|
| | | | | | | Switzerland\|Federal Office of Public Health, Switzerland | |
| 43. | The ESTxENDS Trial: Pulmonary Function Substudy | Completed | Smoking Cessation\|Pulmonary Disease | OTHER: ENDS (vaporizer/e-cig) and smoking cessation counseling\|OTHER: Smoking cessation counseling | University of Bern | Swiss National Science Foundation\|Bernese Lung League\|Clinical Trial Unit Grant of the Inselspital Bern | Interventional |
| 44. | The ESTxENDS Trial-Substudy on the Effects of Using Electronic Nicotine Delivery Systems (ENDS) on Depression | Completed | Smoking Cessation\|Depression | OTHER: ENDS (vaporizer/e-cig) and smoking cessation counseling\|OTHER: Smoking cessation counseling | University of Bern | University of Lausanne\|University of Geneva, Switzerland\|University of Zurich\|State Hospital, St. Gallen\|Swiss National Science Foundation\|Krebsforschung Schweiz, Bern, Switzerland\|Federal Office of Public Health, Switzerland | Interventional |
